# Bedside or not bedside: Evaluation of patient satisfaction in intensive medical rehabilitation wards

**DOI:** 10.1371/journal.pone.0170474

**Published:** 2017-02-07

**Authors:** Christophe Luthy, Patricia Francis Gerstel, Angela Pugliesi, Valérie Piguet, Anne-Françoise Allaz, Christine Cedraschi

**Affiliations:** 1 Division of General Medical Rehabilitation, Geneva University Hospitals & University of Geneva, Geneva, Switzerland; 2 Division of Quality of Care, Geneva University Hospitals, Geneva, Switzerland; 3 Division of Oncology, Geneva University Hospitals, Geneva, Switzerland; 4 Division of Clinical Pharmacology and Toxicology, Geneva University Hospitals & University of Geneva, Geneva, Switzerland; Public Library of Science, FRANCE

## Abstract

**Background:**

Concerns that bedside presentation (BsP) rounds could make patients uncomfortable led many residency programs to move daily rounds outside the patients’ room (OsPR). We performed a prospective quasi-experimental controlled study measuring the effect of these two approaches on patient satisfaction.

**Methods:**

Patient satisfaction was measured using the Picker questionnaire (PiQ). Results are expressed in problematic percentage scores scaled from 0 = best-100 = worst. During three months, 3 wards of a 6 ward medical rehabilitation division implemented BsP and 3 control wards kept their usual organization of rounds. In total, 90 patients of each group were included in the study and completed the PiQ.

**Results:**

Socio-clinical characteristics were similar in both groups: mean age = 67 years (SD = 13), mean Charlson comorbidity index = 8.6 (2.4); mean length of stay = 22 days (12). During their stay, patients in the BsP units had a mean of 14.3 (8) BsP rounds and 0.5 (0.8) OsPR; control patients had a mean of 0.9 (0.7) BsP and 14.8 (7.3) OsPR (p<0.0001). Patients in BsP units reported lower problematic scores regarding coordination of care (39% vs 45%, p = 0.029), involvement of family/friends (29 vs 41%, p = 0.006) and continuity/transition (44% vs 54%, p = 0.020); two questions of the PiQ had worse scores in the BsP: trust in nurses (46.7% vs 30 %, p = 0.021) and recommendation of the institution (61.1% vs 44.4%. p = 0.025). No worsening in dimensions such as respect for patient preferences was seen.

**Conclusions:**

BsP rounds influenced the patient-healthcare professionals’ encounter. These rounds were associated with improved patient satisfaction with care, particularly regarding interprofessional collaboration and discharge planning.

## Introduction

Time dedicated to ward rounds (between 3 and 12 minutes per patient) varies according to the clinical setting [1]. In any case, these rounds constitute an important part of the healthcare team workday [2]. Bedside rounds is a manifold task as it aims to gather and synthesize information about patients, to transmit interdisciplinary information, to build a shared project of care and decision making with the patient[3]. These tasks require medical knowledge, communication skills, both with patients and with multidisciplinary healthcare teams, as well as a capacity to deal with time and organizational constraints [4].

Bedside rounds have given rise to a series of concerns regarding the stress they may induce in patients, the confusion generated by use of medical jargon, confidentiality breaches and loss of efficacy for the healthcare team [5]. Globally, these concerns were eased by the results of a few studies showing that strategies used by experienced bedside teachers can be used for faculty development aimed at promoting bedside rounds [5–7]. However, collective bedside rounds remain a challenging activity for the healthcare professionals who often prefer to conduct these rounds outside of the patient’s room, arguing for lack of time, organisational constraints, insufficient faculty experience and training in conducting bedside teaching rounds, and perceived or actual decreased efficiency in daily time-management due to the process of team walk- rounds with computerised charts [5,8–9]. Studies are heterogeneous both in terms of design and setting and thus preclude generalisation regarding benefits and difficulties of bedside rounds.

The literature shows that bedside rounds are associated with increased patient satisfaction when these rounds are compared to visits conducted outside of the patient’s room (e.g. in the conference room). Such results have been evidenced in various settings (internal medicine, pediatrics, obstetrics), in various countries and cultural backgrounds [10–16]. Few studies have shown that, on the contrary, patients favoured rounds outside the patient’s room [17–19].

Patient Reported Outcome Measures (PROMs) assess the quality of care delivered to patients from the patient’s perspective [20]. Indeed, although clinicians can make objective observations of signs, impairment and disability, only patients can report on their symptoms, needs and overall satisfaction with care. These dimensions are also related to other endpoints, such as clinical safety and effectiveness [21]. We conducted a controlled study with one of the most widely used multidimensional questionnaires for inpatient satisfaction, validated in national and international settings [22] which assess satisfaction with both process and outcomes of care, in a general medical rehabilitation ward.

## Methods

### Design and participants

This is a prospective quasi-experimental controlled study conducted at a 96-bed university based general medical rehabilitation ward comprising 8 units of 12 patients each with a mean of 2.4 patients/room. The ward is part of a 1,200-bed urban public and teaching hospital which is the major primary care hospital for the area and is devoted to general medical rehabilitation and psychosocial care with a specific emphasis on comprehensive active rehabilitation and multidisciplinary treatment. Patients are either transferred from acute care wards (about 2/3) or directly admitted from the emergency room (about 1/3) in any one of the eight units of the ward. Median and mean length of stay are 16 and 21 days, respectively. A vast majority of the patients are discharged home, 7–8% of the patients die, and only very few (1–2%) require a definitive institutionalization. The day shift includes eight residents (resident-patient ratio = 1:12), eight medical trainees (medical trainees-patient ratio: 1:12), four senior residents (senior residents-patient ratio = 1:24), two attending physicians (attending physicians-patient ratio = 1:48), 59 nurses (nurse-patients ratio = 1:5). From the day of admission, every patient is seen at least once a day by the physician in charge on a one to one basis. Depending on the requirements of the patient’s clinical status, the resident, the senior resident and the nurses may visit the patient again to complete clinical examinations, interviews, plan discharge, and meet the patient’s proxies. Aside from this one to one patient-physician encounter, daily rounds are carried out, with the participation of one resident, one medical trainee and one nurse and lasting, in general, for two hours every morning. Once a week, a grand round also includes the senior resident and an attending physician or the medical head of the department. The staff was informed of the purpose of the study and the ward was divided into two groups, i.e., one group of four units was assigned to conduct bedside rounds and the other group of four units was assigned to rounds as usual, whether bedside or outside of the patient’s room. The rest of the daily clinical activity remained unchanged in all the units of the ward. As all eight units have the same function and resources, patients are admitted indifferently in either of these units. As the rehabilitation ward is physically located on two different floors of the hospital, for the sake of simplicity, the four units on the second floor were allocated to the intervention group and those four on the third floor were allocated to the control group.

### Pilot phase

To assess the natural conditions in the ward, a pilot phase was conducted with consecutive patients discharged from the eight units of the ward during a two-month period. The assessment used the same measurements as those used in the subsequent controlled study.

### Intervention and control conditions

We developed discreet mutually exclusive categories for location of rounds (bedside or hallway). The intervention group was asked to conduct the round at the patient’s bedside. The rationale for this explicit request was the importance of increasing time spent with the patients. This request was inserted within the global policy of the rehabilitation ward that emphasized the importance of explaining therapeutic goals of the hospital stay to improve patients’ participation in the care process and reinforcing multidisciplinary collaboration. No formal instruction or checklist was provided in either group concerning the contents or the course of the rounds. The control group conducted the rounds either at the patient’s bedside or in the hallway as they considered appropriate. All members of the staff were informed that patient satisfaction would be anonymously assessed before discharge for those having signed an informed consent form. As this was not a human subjects research but an institutional quality improvement study, it was exempt from the Institutional review board of the Geneva University Hospitals. All patient data was anonymised. All members of the staff were informed that patient satisfaction would be anonymously assessed before discharge for those having signed an informed consent form (all recruited patients did sign).

### Measurements

Socio-demographic and clinical characteristics of the patients were extracted from the medical charts. Charlson index was used to assess comorbidities [23], and length of stay was considered as well. As for patient satisfaction, we used the Picker questionnaire [22]. The 44-item Picker Patient Experience is a self-administered questionnaire that measures eight specific aspects of inpatient experience: emotional support, respect for patient preferences, involvement of family and friends, information and education, information specific to surgery, continuity and transition, coordination of care, physical comfort and one general satisfaction dimension: overall impression. It has been shown that this questionnaire has high degrees of both face and construct validity as well as internal reliability consistency [22].A randomized trial of four patient satisfaction questionnaires demonstrated that the Picker questionnaire was the most likely to cover important areas regarding hospital stay and be the least difficult to understand as well as being the best designed questionnaire [24]. Results of the PiQ are expressed in problematic percentage scores scaled from 0 = best to 100 = worst [22]. Additional questions routinely administered in patient satisfaction surveys in our institution were also included. These questions aim to respond to specific concerns of the institution and are inserted within the dimensions of the Picker questionnaire (“could you identify a nurse in charge of your treatment during your stay?”, “How well was your discharge organized?” and “Did you get the impression that care was withheld to reduce costs?”). The same scaling was used for these questions also.

### Primary outcome

Patient satisfaction as measured with the Picker questionnaire and additional patient satisfaction items described above.

### Sample size

The assumptions made to calculate the study sample size considered that a relative decrease of 15% in problematic values in the satisfaction of the patients included in the intervention group to be significant. We calculated that a group of 90 patients per group would reach enough power to detect a 15% decrease with a significance level of 0.05.

### Data management and analysis

Patient characteristics were compared using chi-square tests for categorical variables and Kruskall-Wallis tests for continuous variables. As inter class correlation coefficients of multilevel mixed models were close to zero, using a two level approach with unit as the grouping variable, standard modeling was used. Analyses of differences in individual items of satisfaction were carried out with chi-square tests and the 9 satisfaction dimensions between groups were assessed with one-way ANOVA tests. All analyses were performed using Stata release 12 (Stata Corporation, College Station, Texas).

### Ethics approval

As this was not a human subjects research but an institutional quality improvement study, it was exempt from the Institutional review board of the Geneva University Hospitals. All patient data was anonymised. All members of the staff were informed that patient satisfaction would be anonymously assessed before discharge for those having signed an informed consent form (all recruited patients did sign).

## Results

### Patients’ characteristics

Ninety consecutive patients for the pilot phase were included between May and June 2012 and 180 consecutive patients for the controlled study were included between December 2012 and March 2013. All of them gave their signed informed consent to participate in the study. Patients had a mean age of 67 years (SD = 13 years); women and men were equally distributed (50.4% vs 49.6%); level of education mostly included professional diploma (55%) and high school (20%); degree of co-morbidity was fairly high (Charlson median = 8.6; ± 2.4) and mean length of stay amounted 22 days (SD = 12). Most (70%), patients were discharged home and a minority (30%) were transferred to another long-term or convalescence care structure. There were no differences in patient characteristics between the intervention and control groups or between the pilot and the control phases.

### Patient satisfaction: Pilot phase

In this group (N = 90), visits were mainly hallway and only rarely conducted at the bedside. The results for patient satisfaction were very similar to those obtained in the control group in the subsequent phase of the study (data not shown).

### Patient satisfaction: Intervention vs control group

During their stay, the patients included in the intervention group (N = 90) received a mean of 14.3 (SD = 8) bedside visits and 0.5 (SD = 0.8) hallway visits; the 90 patients included in the control group received a mean of 0.9 (SD = 0.7) bedside visits and a mean of 14.8 (SD = 7.3) hallway visits (p<0.001). As for satisfaction, 10 out of the 44 questions of the Picker questionnaire showed significant between-group differences in favour of the intervention group (Table 1). The decrease of problematic values in favour of the intervention group evidenced a reduction of problematic scores ranging from 9% to 26% (Table 1).

**Table 1:** Between-group differences in patient satisfaction as measured by the Picker inpatient satisfaction questionnaire.

Picker questions	Intervention group problematic score* (%) N = 90	Control group problematic score (%) N = 90	p value**
Problem identifying doctor in charge	2.2	28.9	<0.001
Doctor–nurse teamwork not good	2.2	11.1	0.017
Doctor didn’t discuss anxieties or fears	26.7	45.6	0.008
Nurses didn’t discuss anxieties or fears	30	47.8	0.014
Didn’t always have confidence and trust in nurses	46.7	30	0.021
Not easy to find someone to talk to about concerns	28.9	45.6	0.021
Doctor not available	8.9	21.1	0.022
Not sufficiently involved in treatment	54.4	72.2	0.013
Purpose of medication not sufficiently explained	45.6	68.9	0.002
Family not given enough information about condition or treatment	11.1	37.8	<0.001
Family not given information needed to help recovery	33.3	48.9	0.034
Hospital would not be recommended	61.1	44.4	0.025

*Results of the PiQ are expressed in percentage scores scaled from 0 = best to100 = worst

**Pearson’s chi squared statistic

In addition, three dimensions of the Picker patient survey showed a significant improvement in the intervention group, i.e. dimensions relating to treatment coordination, involvement of family and friends, and discharge planning (Table 2).

**Table 2:** Main dimensions of patient satisfaction as measured by the Picker questionnaire for the two groups (N = 90 unless otherwise stated).

Picker Dimension	Intervention group problematic* score %	Control group problematic score %	p value**
Information and education	43.9	44.9	0.794
Coordination of care	39.6	45.2	0.029
Physical comfort	41.6	41.4	0.965
Emotional support	34.2	40.4	0.203
Respect for patient preferences	47.8	54.2	0.095
Information in surgery	43.3 (N = 15)	36.7 (N = 17)	0.531
Involvement of family and friends	28.5	40.7	0.006
Continuity and transition	43.6	53.9	0.020
General impression	16.8	17.5	0.763

* Results of the PiQ are expressed in percentage scores scaled from 0 = best to100 = worst.

**one-way ANOVA

Only two questions of the Picker questionnaire had worse scores in the intervention group (Table 1): those pertaining to trust in nurses and recommendation of the institution. There was no decrease in satisfaction for any dimension and in particular satisfaction in the dimensions of patient preferences and emotional support was the same in both groups. Additional satisfaction items also showed a significant or borderline significant decrease of problematic values in the intervention group (Table 3).

**Table 3:** Between group differences of problematic scores as measured by additional questions.

Additional questions	Intervention group problematic* score (%) N = 90	Control group problematic score (%) N = 90	p value**
Problem identifying nurse in charge	48.9	63.3	0.051
Discharge not well organised	43.3	65.6	0.003
Impression that care was withheld to reduce costs	52.2	71.1	0.015

* Results of the PiQ are expressed in percentage scores scaled from 0 = best to 100 = worst.

**Pearson’s chi squared statistic

## Discussion

This pragmatic study showed that when assigned to conduct rounds as usual, healthcare teams perform a vast majority of visits in the hallway and only few visits at the patient’s bedside. Yet, when requested to conduct bedside rounds, they change their usual clinical practice and the number of visits at the patient’s bedside significantly increases. This change was associated with a significant improvement of patient satisfaction with care, in particular with identification of the treating physician and nurse, the possibility of getting sufficient information about one’s health and treatment, the ability to express one’s worries and fears with the physician or nurse in charge, the opportunity to discuss treatments tailored to the patient’s needs and to use a shared-decision making approach; these elements may contribute to significant improvements in terms of satisfaction with the dimensions of treatment coordination, information and discharge planning. In addition, despite potential breaches in confidentiality or possible use of medical jargon, the bedside rounds group experienced no decrease in the dimension of satisfaction related to feeling respected as an individual. Considering this general picture, it somehow comes as a surprise that recommendation of the hospital was lower in the bedside rounds group than in the control group; however, it is also noteworthy that the additional satisfaction item related to restriction of care due to financial constraints showed that this was less of a preoccupation in this group. This latter result is in line with the absence changes in the hospital setting, i.e. the two groups had similar staffing and number of procedures during the study period.

Our results parallel those of the few studies that have compared bedside rounds with hallway or conference room visits [12,25]. Two controlled studies [13,15] and one randomized controlled trial [16] have shown that patients were more satisfied with in-room case presentations than with out-of-room presentations. Other advantages of bedside visits have been suggested, although not clearly demonstrated, such as better patient understanding of their illness, higher participation in the decision-making process as well as better or more complete information provided by the patient [10,13]. Physician-nurse team bedside visits have been shown to improve various aspects of treatment in intensive care units and in particular pain management and mortality [26–27]. Only one drawback of bedside visits for conducting physicians could be demonstrated in the RCT cited above [16]: residents and medical trainees may have felt less comfortable asking questions and responding to patient’s queries. Other studies have also shown less satisfaction in medical trainees and junior residents with bedside rounds [11,13,15,28]. All residents and nurses participating to the present study were experienced care professionals. As for the residents, they had an average postgraduate training duration of 2 to 4years (in Switzerland, most residents begin their training after medical school in non-university hospitals). These characteristics are similar to those of most teams in university hospitals and this should also allow the resident-nurse team to transmit their collaborative experience to younger faculty with less experience with teaching at the bedside [6,7].This is all the more so in the weekly rounds and the grand rounds including senior residents and attending physicians.

Another possible interpretation of these results may be the discomfort related to changes in practice. From the patients’ standpoint however, the evidence for potential drawbacks in conducting bedside rounds is sparse. Some authors have shown that the use of medical jargon may induce anxiety [8,10]. Furthermore, confidentiality must be preserved and the patient has to be part of the process and be given enough opportunities to participate in the discussion [12].

Centrifugal forces do exist that ward off clinicians from patients while care should be centered on the latter [10]. Time constraints and overabundance of data turn electronic medical charts and paraclinical tests into powerful centers of attention. One possible way to counteract these forces is to present and discuss the patient’s illness and its repercussions in the patient’s presence. From a medical standpoint, conducting bedside rounds also sends a clear message: ‘*medicine is something done with the patient*, *not to the patient*’ [10].

Strategies are available to overcome barriers related to bedside rounds and to aid in preparing for the patient encounter. Firstly, the team should be conscious of how far the patient has been informed or is aware of his/her medical condition; they should also take time to explain the process and aims of bedside rounds and reassure the patient about its course. During the bedside rounds caution should be taken when discussing diagnoses, soliciting the patient’s beliefs and knowledge as well as his/her fears. Another issue of importance is preserving confidentiality when informing and questioning the patient in shared rooms. These aspects are of clear significance as they also belong to patient’s definition of what is a good therapist [29]. This means that the ward round should be prepared in advance, revolve around a checklist, and emphasize dialogue between participants [7,30]. Our study did not include formal assessment of the content of bedside rounds, but other studies have stressed that interaction styles can vary greatly, e.g. in terms of time devoted to clinical data gathering or to patient physical examination [2,31].

The integration of the nurses in the bedside rounds is another issue as in everyday practice it is not customary to discuss work procedures and debrief teamwork performance [30]. The importance of interprofessional collaboration has been stressed, along with the difficulty to demonstrate which elements of its organization and content are central for this collaboration to be truly effective [32–33]. Indeed, in our study, trust in nurses was lower in the intervention group than in the control group. Although we cannot determine the exact cause of this, it could be that poor interprofessional preparation and insufficient allotment of roles prior to bedside visits played a part. These issues highlight the importance of interprofessional education to foster a shared vision and understanding of each other’s roles [34], as well the importance of joint training for better patient care. In addition, the perceived lack of trust in nurses in the intervention group could also have had an influence on hospital recommendation as the intervention group had a higher problematic score as well. Indeed, prior studies have shown the central role of nurse activities on patient satisfaction and hospital recommendation [35, 36].

Much in the same line, the value of role modeling as an informal part of medical training has been stressed, not only between doctors but also when it comes to being open to the views of other health professionals and of patients [37]. Nurses and doctors visiting the patient have different competencies, expectations and languages as well as a different relationship with the patient that needs to be fine-tuned before visiting the patient. Young residents and nurses may not be comfortable with bedside rounds and may thus benefit from participating in the rounds or grand rounds with the senior residents and attending physicians who can provide positive role modeling in so far as they share their experience in teaching, liaison with the staff and emphasize the importance given to the patient-therapist relationship [38].

### Study strengths and limitations

Methodological strengths included prospective patients’ evaluation and controlled measures with validated instruments during both pilot and intervention periods. The pilot data collection (pre-intervention) performed in all units of the ward allowed for establishing that no other unit-level changes contributed to changes in clinical practice at the time of the intervention period in the control units; and that pilot and intervention patient cohorts were equivalent (sociodemographic, co-morbidity index and length of stay). Neither the intervention nor the control group were devoid of the other setting, i.e. not all intervention group conducted bedside only and not all control group conducted solely hallway visits, but interestingly, the analysis showed that the results of the control group showed the opposite proportion of bedside vs. hallway rounds as compared to the intervention group were bedside rounds were specifically requested. Because this was a pragmatic study, we decided to keep all results observed in each group as the instruction in the control group was to conduct the rounds either at the patient’s bedside or in the hallway as they considered appropriate [39]. While this was a single-site study, the sample size exceeded requirements to detect significant differences in patients’ satisfaction. As for the possible generalization of our results, medical organization and patients’ characteristics were very similar to those of other general in-patient medical rehabilitation wards of university hospitals in Switzerland. The Hawthorne effect may have influenced our findings; although physicians were not provided with explicit project information until data had been collected, there may have been heightened awareness associated with patients’ satisfaction during the intervention period. As a quality improvement initiative, the intervention provided healthcare teams with relevant clinical information to facilitate their commitment with the project. Healthcare teams were therefore aware of the main study aim (i.e.to improve ward rounds efficiency and patient satisfaction) and this may have influenced their behaviour during the intervention period, but it is not likely that this is a source of significant bias as both groups were informed in the same manner. It is noteworthy however, that the intervention group received no formal tutoring on conducting interdisciplinary bedside rounds, they were only informed of the need to increase time spent with the patients and further stress the therapeutic goals of their hospital stay. Patients were not asked whether they felt bedside rounding violated their confidentiality; such an investigation may be of interest; however, in this study, despite potential breaches in confidentiality, the bedside rounds group experienced no decrease in the dimension of satisfaction related to feeling respected as an individual. Care providers were not asked for feedback on their experience with either type of round. These sources of information would have been of interest to better understand and improve the rounding process.

## Conclusion

Bedside visits are an essential part of inpatient care that contributes to increased patient satisfaction with better family involvement, care coordination and transition of care. However, bedside visits can also be associated with worse scores on certain items such as trust in nurses and hospital recommendation. Even though our study did not explore the reasons for this, it could be that insufficient preparation of the healthcare team and lack of allotment of roles prior to visits contributed to this result. Further studies need to be conducted to explore these aspects and to determine if better preparation and role assignment will lead to increased confidence. Nevertheless, the challenge remains in improving interdisciplinary education and training and in developing quality metrics specific to bedside visits that emphasize and enhance teamwork.
